# Investigating associations between nematode infection and three measures of sociality in Asian elephants

**DOI:** 10.1007/s00265-022-03192-8

**Published:** 2022-06-24

**Authors:** Carly L. Lynsdale, Martin W. Seltmann, Nay Oo Mon, Htoo Htoo Aung, UKyaw Nyein, Win Htut, Mirkka Lahdenperä, Virpi Lummaa

**Affiliations:** 1 Natural Resources Institute, Helsinki, Finland; 2 Helsinki Institute of Life Science, University of Helsinki, Helsinki, Finland; 3 Faculty of Biological and Environmental Sciences, University of Helsinki, Helsinki, Finland; 4 Department of Biology, University of Turku, 20014 Turku, Finland; 5 Department of Animal Science, University of Veterinary Science, Yezin, Myanmar; 6 Myanma Timber Enterprise, Ministry of Natural Resources and Environmental Conservation, Yangon, Myanmar; 7 Department of Public Health, University of Turku and Turku University Hospital, 20014 Turku, Finland; 8 Centre for Population Health Research, University of Turku and Turku University Hospital, 20014 Turku, Finland

**Keywords:** Host-parasite dynamics, Infection costs, Long-lived mammal, Parasite ecology, Social behaviour

## Abstract

**Abstract:**

Frequent social interactions, proximity to conspecifics, and group density are main drivers of infections and parasite transmissions. However, recent theoretical and empirical studies suggest that the health benefits of sociality and group living can outweigh the costs of infection and help social individuals fight infections or increase their infection-related tolerance level. Here, we combine the advantage of studying artificially created social work groups with different demographic compositions with free-range feeding and social behaviours in semi-captive Asian elephants (*Elephas maximus*), employed in timber logging in Myanmar. We examine the link between gastro-intestinal nematode load (strongyles and *Strongyloides* spp*.*), estimated by faecal egg counts, and three different aspects of an elephant’s social world: individual solitary behaviour, work group size, and work group sex ratio. Controlling for sex, age, origin, time since last deworming treatment, year, human sampler bias, and individual identity, we found that infection by nematodes ranged from 0 to 2720 eggs/g between and within 26 male and 45 female elephants over the 4-year study period. However, such variation was not linked to any investigated measures of sociality in either males or females. Our findings highlight the need for finer-scale studies, establishing how sociality is limited by, mitigates, or protects against infection in different ecological contexts, to fully understand the mechanisms underlying these pathways.

**Significance statement:**

Being social involves not only benefits, such as improved health, but also costs, including increased risk of parasitism and infectious disease. We studied the relationship between and three different sociality measures—solitary behaviour, group size, and the proportion of females to males within a group—and infection by gut nematodes (roundworms), using a unique study system of semi-captive working Asian elephants. Our system allows for observing how infection is linked to sociality measures across different social frameworks. We found that none of our social measures was associated with nematode infection in the studied elephants. Our results therefore suggest that here infection is not a large cost to group living, that it can be alleviated by the benefits of increased sociality, or that there are weak infection–sociality associations present which could not be captured and thus require finer-scale measures than those studied here. Overall, more studies are needed from a diverse range of systems that investigate specific aspects of social infection dynamics.

**Supplementary Information:**

The online version contains supplementary material available at 10.1007/s00265-022-03192-8.

## Introduction

In social species, group living and social behaviours can promote reproduction and survival through numerous pathways, including increased offspring survival, increased access to potential mates and resources, protection from predation, and increased health via social support (reviewed in Cantor et al. 2021). However, the same mechanisms that offer these benefits—frequent social interactions, close proximity to conspecifics, and group density—also present costs, such as increased competition and conflict (Alexander 1974; Krause and Ruxton 2002) and risk of disease and parasite infection (McEwen 2012; Hawley et al. 2021). Different components of sociality affect parasite load in various ways. For instance, group size is positively related to intensities of non-mobile parasites, but negatively to intensities of mobile parasites (Patterson and Ruckstuhl 2013). When investigating host–parasite interactions, it is important to consider the three main components of disease, called the disease triangle: the host, the environment, and the pathogen/parasite (Scholthof 2007). Individual host characteristics such as age (Lynsdale et al. 2020) and sex (Hillegass et al. 2008), as well as behaviour and social status (Hawley et al. 2011; Keiser et al. 2016), can relate to transmission and infection risk. In addition, external factors, such as season or weather conditions, influence sickness behaviour and infection dynamics for environmentally transmitted parasites (Owen-Ashley and Wingfield 2006; Rödel and Starkloff 2014). Finally, parasites can influence host social behaviour to promote transmission of parasites from the infected to new hosts (Moore 2002; Hawley et al. 2021).

The “classic” view of social–infection dynamics is that sociality is a main driver of infections (Rifkin et al. 2012; Patterson and Ruckstuhl 2013). This view has substantial support within the literature: Higher numbers of social contacts and frequent social interaction are generally linked to increased infection (Loehle 1995; Schmid-Hempel 2017), and the reverse for increasingly solitary behaviour. A recent review on over 200 associations between individual social network measures and parasite load has shown that, within individuals, social behaviour leads to an increased risk of parasite infection (Briard and Ezenwa 2021). However, a growing body of theoretical and empirical studies challenges the assumption that infection risk and social behaviour or group size always co-vary positively (Kappeler et al. 2015; Ezenwa et al. 2016). This “enhanced” view suggests that the health benefits of sociality and group living can outweigh the costs of infection and help social individuals resist or tolerate parasites and other infectious disease (Ezenwa et al. 2016). Several studies have demonstrated that positive social interactions can be related to lower infection risk and lower infestation with gastrointestinal parasites. Social support by known group members and strong social bonds with opposite-sex partners can reduce parasite infestation (Rödel and Starkloff 2014; Müller-Klein et al. 2019), though this effect can depend on various factors such as environmental conditions or pathogen-specific transmission routes (Balasubramaniam et al. 2016). The encounter-dilution effect describes the potentially positive effects of group living with regard to costs of parasite infection (Mooring and Hart 1992), where group members experience protection from parasites by diluting the risk of being preyed on by ectoparasites or vector species (Mooring and Hart 1992; Patterson and Ruckstuhl 2013). Additionally, social living per se can reduce the negative effects of parasite infestation and larger group size can mitigate the costs of infection with ectoparasites for group members (Almberg et al. 2015). Furthermore, the positive effects of group size have been proposed and described for endoparasites and other infectious diseases. Although re-infection with gastrointestinal nematodes is more likely for individuals in larger social groups, infected individuals benefit from larger intake of energy, which offsets main costs of nematode infection (Ezenwa and Worsley-Tonks 2018). Interestingly, the link between group size and parasite loads is often species-specific and related to other social measures. In African bovids, a positive correlation between group size and parasite infection was found, but this was only evident for relatively host-specific parasites and for hosts living in stable groups (Ezenwa 2004).

The relative infection costs versus sociality benefits of group living should be investigated under various contexts. One interesting, but understudied, factor where individual host characteristics and social group properties can interact on infection dynamics is the sex ratio of the group. Individual sex represents a dichotomy in social behaviour and immunity for many mammal species, e.g. males exhibit solitary or nomadic behaviour more often than females (Lawson Handley and Perrin 2007), which could differentially affect transmission dynamics. Life-history theory dictates that differential selection pressures, prioritising reproduction for males and longevity for females, drive sex-specific differences in resource-allocation trade-offs between immunity and reproduction (Trivers 1972; Stearns 1992; Norris and Evans 1999). Hamilton and Zuk (1982) first proposed parasitism as a mediator for these trade-offs, which can be maintained by either the boosting or regulatory effects of oestrogen and testosterone respectively on individual immune function (Folstad and Karter 1992), alongside behavioural traits that lead to differences in transmission and exposure (Patterson and Schulte-Hostedde 2011). Consequently, parasite infection intensity (i.e. parasites per host) is often higher for adult males, in comparison to females, within mammal populations (Giery and Layman 2019). Sex effects and group size effects are well investigated, but sex ratio effects on parasite infection have rarely been studied in natural systems despite the clear potential for it to influence infection dynamics within group-living species.

Asian elephants (*Elephas maximus*) are an interesting species to address those questions because they are a long-lived and highly social species, with life-history traits similar to several primate and cetacean species such as humans and killer whales (*Orcinus orca*). Studies on elephants can therefore help enable generalizations across larger highly social mammals in understanding the link between sociality and parasite infection. In the wild, Asian elephants form complex social organisations, predominantly existing in matriarchal herds of matrilineal female relatives and juveniles, with males leaving the herd and becoming nomadic upon sexual maturity (Sukumar 2003). Furthermore, elephant society provides benefits such as predator defence, the transfer of social knowledge, and alloparental care of offspring (Wittemyer et al. 2005). However, elephants’ high sociality also imposes costs, such as increasing costs of philopatry for older individuals and increased resource competition (Wittemyer et al. 2005), as well as facilitating the spread and persistence of parasite infections (Hawley et al. 2021). In conditions where the social setting of large long-lived mammals is artificially modulated by humans, the link between sociality and infection is less well understood. Gastro-intestinal nematodes are among the most abundant internal parasites found in Asian elephants (Fowler and Mikota 2006; Lynsdale et al. 2020), are an important driver of elephant mortality (Lynsdale et al. 2017), and are linked to reduced elephant health and immunity (Santos et al. 2020). However, the results regarding the link between parasite load and sociality in elephants are inconsistent (Vanitha et al. 2011; Abhijith et al. 2018) and warrant further investigation. Both individual host characteristics and social group properties are important predictors of parasite infection; however, the outcome of these predictions can vary depending on the classic or expanded view of the parasite-related costs and benefits of sociality (Ezenwa et al. 2016). There is a need for more empirical studies to expand the range of investigated species and systems for animals, as this will ultimately help us improve our understanding of how individuals balance the cost of parasite exposure on the one hand and the benefits of increased parasite tolerance on the other hand in the wild social living animals (Ezenwa et al. 2016).

Here, we take advantage of a unique dataset on semi-captive timber Asian elephants from Myanmar to investigate the link between sociality and parasite infection in a long-lived and highly social mammal. This population is ideal for studying this relationship because their age-specific survival rates and social behaviours resemble those of wild elephants compared to those of fully captive individuals (Seltmann et al. 2018; Clubb et al. 2008; Hayward et al. 2014; Lahdenperä et al. 2016; Chapman et al. 2019; Lynch et al. 2019). In addition, the Myanma Timber Enterprise (MTE) has maintained extensive logbooks on each individual, which allow tracking individual elephants’ life events, such as illness and health treatments, and provide detailed data on group compositions and friendship networks. In addition, we capitalize on the longitudinal data on parasite infection already existing in this system (Lynsdale et al. 2017, 2020), which is highly important for gaining a reliable quantification of infection dynamics as opposed to cross-sectional studies and opportunistic sampling. In these semi-captive elephants, nematode infections happen via faecal–oral horizontal transmission, which is the same route found for wild elephant populations. Adult worms live and reproduce in the gut and gastro-intestinal tract, with eggs expelled with elephant faeces (Fowler and Mikota 2006). Hence, routes of transmission of, and exposure to, local pathogens are potentially similar to those experienced by wild systems compared to fully captive systems, given the studied semi-captive individuals live in their natural habitat and express nocturnal free-roaming behaviours. Myanmar timber elephants are grouped together in mixed-sex units of approx. 4–12 individuals that work within the same forest area, overall spending at least ~ 4–8 h/day together in their working groups, for over 9 months of the year. Individuals therefore spend more time during diurnal hours within close proximity of other group members than non-group conspecifics, occupying shared physical environments where all group members can forage, defecate, and interact. This is important considering the faecal–oral environmental transmission of strongyle and *Strongyloides* spp. nematodes between elephant hosts (Fowler and Mikota 2006), and that Asian elephants display trunk touches around and inside other conspecifics’ mouths as a form of reassurance behaviour (Plotnik and de Waal 2014). Therefore, our study system offers a unique opportunity to study the relationship between sociality and later parasite infection in a semi-experimental way, as elephants live in mixed-sex and age groups with different demographic compositions.

Our work offers data on how different measures of sociality are related to later infection in known individuals of a large, long-lived mammal, which usually roams over long distances in the wild and is therefore challenging to investigate in such detail under fully wild conditions. We use three measures of sociality and investigate their links to subsequent infection by nematode parasites. More specifically, we (1) investigate if engaging in regular social interactions with conspecifics or being solitary (individual solitary behaviour) is linked to later nematode load, measured as faecal egg counts. As wild Asian elephants exist in either strongly associated female family units, or nomadic males or loosely associated male bachelor groups, we therefore expect subsequent differences in group mitigation of infection to arise from natural sex-specific social frameworks. Specifically, we expect females to gain social benefits which protect against infection, such as elevated health and condition from increased social contact and group living, and for males to minimise infection through increased distance from, and less frequent contact with, other potential infective hosts. Transmission-related costs for adult females should be offset by benefits of higher social interaction, but not for adult males that would otherwise incur lower transmission costs from predominantly solitary lifestyles. Therefore, we predict that solitary females and social males in our study sample exhibit higher nematode loads than social females and solitary males. In addition, we (2) investigate how group size is related to nematode load. In our system, larger group size represents potential for increased nematode transmission due to higher densities of (potential) host feeding and defecating within the same habitat patches, alongside more frequent close, physical interactions, as well as improved individual health, linked to increased social interaction and social support, which may help mitigate or offset the costs of infection. We thus expected a weak but overall positive effect of group size, with individuals in larger groups yielding higher nematode load. Furthermore, we (3) studied the link between the sex ratio of the work group and nematode load. We predicted that elephants in groups that have more males than females, hence in groups with a male-biased sex ratio, show higher levels of parasite infection. In many mammals, males show higher levels of parasite infection than females (Wilson et al. 2002) and there is a need to investigate potential sex effects in the social context in which we find these elephants.

Generally, it is important to expand our understanding of the link between different social measures and parasite infection by expanding the available empirical evidence for those relationships for different species living under different conditions. This can help disentangle the complex associations of sociality and infection and the contradictory results found in previous studies, and to generate a more holistic view of a very topical problem.

## Methods

### Study population

The working timber elephants of Myanmar (*n* ~ 3000) make up the largest remaining semi-captive population of this species (Mar 2007; Hedges et al. 2018). The elephants work as draught animals in logging camps during the day alongside an elephant caretaker or ‘mahout’, but freely roam, forage, and interact with wild and other semi-captive conspecifics at night in surrounding forest habitat (Gale 1974). The current abundance and distribution of wild elephants in Myanmar are not well studied (Leimgruber et al. 2011; Hedges et al. 2018). Myanmar’s wild elephant population is estimated to be fewer than 2000 individuals (Leimgruber et al. 2011), and the chances for encounters between wild and semi-captive elephants are probably low. Though semi-captive elephants roam freely at night, they usually do not leave the wider vicinity of their timber camps. The semi-captive population is centrally managed by MTE, which mark each animal with a unique identification (ID) number on their haunches allowing for reliable recognition of different individuals. MTE staff also keep detailed records in individual log books, on e.g. elephant date of births (if captive born) or capture (if wild caught), location, maternal lineage, disease history, and treatment history, throughout an elephant’s lifetime. Subsequently, MTE maintain longstanding records on Asian elephant life-history and health which are now digitised into an electronic database, allowing for accurate sampling of individuals of known age.

Trained MTE veterinarians are responsible for the basic upkeep of the elephants, and predominantly treat wounds and other working injuries. Vets are also charged with administering anthelmintic drugs (ivermectin and albendazole) approximately twice a year in accordance with state regulations as a blanket treatment rolled out across all elephants within treated camps, irrespective of their level of infection. Treatment is administered either subcutaneously (1 ml/100 kg elephant body weight), or orally (10 mg/100 kg body weight for ivermectin and 750 mg/100 kg body weight for albendazole), in line with equine guidelines. Exact dates of anthelmintic treatment are recorded onsite on the day of deworming in each animal’s logbook.

The entire population is distributed across Myanmar, grouped into mixed-sex working units comprising individuals of mixed ages. Adults enter the workforce at approximately 17 years old and remain until retirement (usually around 55 years of age), with workload set by regulations on haulage ability and elephant age (Mar 2007). Elephants work only during the cold (November–February) and monsoon (June–October) seasons, and are rested in the hottest, driest months. Pregnant mothers are rested from halfway through their pregnancy (11 months), and for approximately 1–2 years following parturition where they are used for light baggage work, although calves remain nearby ‘at heel’ until they are weaned and can suckle as needed (Gale 1974). Following weaning and taming (at approx. age 5 years), young elephants either return to their natal group or are relocated away from their mothers. Overall, the elephants spend approx. 4–8 h/day, during diurnal hours, working and interacting with the other members of their designated groups, throughout their ~ 40-year working life.

### Sample

In total, we sampled 71 focal individuals (total no. of samples = 130 including repeated measures, no. of measures per individual = 1–6, mean = 2), all working within the Kawlin logging agency in the Sagaing Division. Our study population included 45 females (91 samples) and 26 males (39 samples), ranging in age from 10–62 years of age (mean = 26 years, median = 16 years) at the time of sampling, and of which 58 were captive born and 13 were wild caught. It was not possible to record data blind because our study involved focal animals in the field.

### Sociality data collection

We investigated how infection was associated with three specific aspects of elephant sociality: individual solitary behaviour, group size, and group sex ratio. First, in order to assess an individual’s direct social interactions with conspecifics (individual solitary behaviour), we extracted information from social questionnaires given to elephant handlers (mahouts) regarding whether each mahout classed their working animal as solitary (does not interact with other elephants) or social (interacts with other elephants). Mahouts can spend as long as 16 years with the same individual within this sample (Crawley et al. 2019), and thus develop an excellent knowledge of their animal and its behaviour. Questionnaires were carried out locally at field sites during the hot season (March – May) between 2014 and 2018. Next, using the same questionnaire data and recordings by veterinarians, we recorded the overall size of the working group of focal individuals at the time of sampling, only considering the number of adults present. Finally, we determined the sex ratio of the focal individual’s work group by calculating the proportion of females in a group, excluding calves.

### Faecal sampling and nematode quantification

We collected 4.5 g of fresh faecal samples (*n* = 130) from the 71 elephants following a standardized sampling method for our sample population (Lynsdale et al. 2015). Samples were collected within 66 days latest following social data collection, but still within the hot season of March–May. The majority of FECs were collected on the same day as the social data collection was conducted (102/130 measures, ~ 78% of the total sample) and 1 measure was collected the following day. A further 16 FECs were collected within approximately 4–5 weeks after social data collection (25–37 days, ~ 12% of the total sample). Finally, 11 FECs were collected over 5 weeks after social data collection (at exactly 66 days, ~ 8% of the total sample). For each sample, we carried out a faecal egg count (FEC) following the special modification of the McMaster method (MAFF 1986), as in Lynsdale et al. (2020), using compound microscopes with × 10 optical zoom and × 10 magnification. We identified ova microscopically to the lowest taxonomic unit via identification of size, morphology, and developmental stage (Taylor et al. 2007; Bowman 2014). We obtained a quantified estimate of nematode load by multiplying FECs by the dilution factor (10) to convert counts into measures in eggs per gram (epg) of faeces. While FECs are a widely recognised measure of observable parasite load in veterinary and ecological studies, no study has yet provided empirical data on how FECs vary with ultimate measures of infection, e.g. intestinal worm counts, in elephants, and FECs may not account for, e.g. immature larvae, variation in shedding rates of female worms, prepatent periods, and non-reproductive individuals. As such, FECs should be regarded as a reliable estimate of the extent of infection (i.e. approximate load), rather than an exact sum of the total infective agents within a host; see Lynsdale et al. (2020) for further detail.

### Statistical analysis

We analysed the association between the social landscape and subsequent infection by nematode parasites, as estimated via FECs, in our study population using three separate generalised linear mixed-effects models. All analyses were carried out in *R* 4.0.3 (R Core Team 2020) using *glmmTMB* (Anderson and Winter 2020), with untransformed FECs as the response term, and fitted to a negative binomial error structure (*nbinom2*), to account for the overdispersed distribution of FECs based on the mean–variance relationship of the data (Lynsdale et al. 2020). Each of our three models contained one separate univariate predictor pertaining to our sociality measures: sociality (binary, social/solitary), working group size (continuous), and working group female:male sex ratio (continuous). All models started with the same fixed covariates accounting for elephant age (continuous, years)—included to the highest significant polynomial level, sex (two-level factor, male/female), origin (two-level factor, captive born/wild caught), sample year (five-level factor, one level for each year 2014–2018), human sampler bias (three-level factor, one for each sampler who collected data), and time since last deworming treatment prior to sampling (continuous, days). However, models including year and sampler bias did not converge. Hence, we excluded sampler bias from further analyses because in models including only year and sampler bias, year was significant, whereas sampler bias was not. We also included two random factors to account for repeated measures from the same individuals (elephant ID), and from individuals located within the same working group (group ID). We tested all fixed covariate and random terms using likelihood ratio tests (LRTs), comparing starting models to replicates without each singular term in turn. Finally, as Asian elephants display clear sexual dimorphism in social structure and behaviours in the wild, we tested whether sociality–infection dynamics differed between males and females by including an interaction between sex and the social measure included (solitary behaviour, group size, group sex ratio) after excluding other non-significant covariates. Final models retained only significant confounding covariates, as well as our social terms of interest (sociality, working group size, and sex ratio). We checked models for goodness of fit using residual diagnostic checks with the *DHARMa* package (Hartig 2020).

## Results

**Fig. 1:**
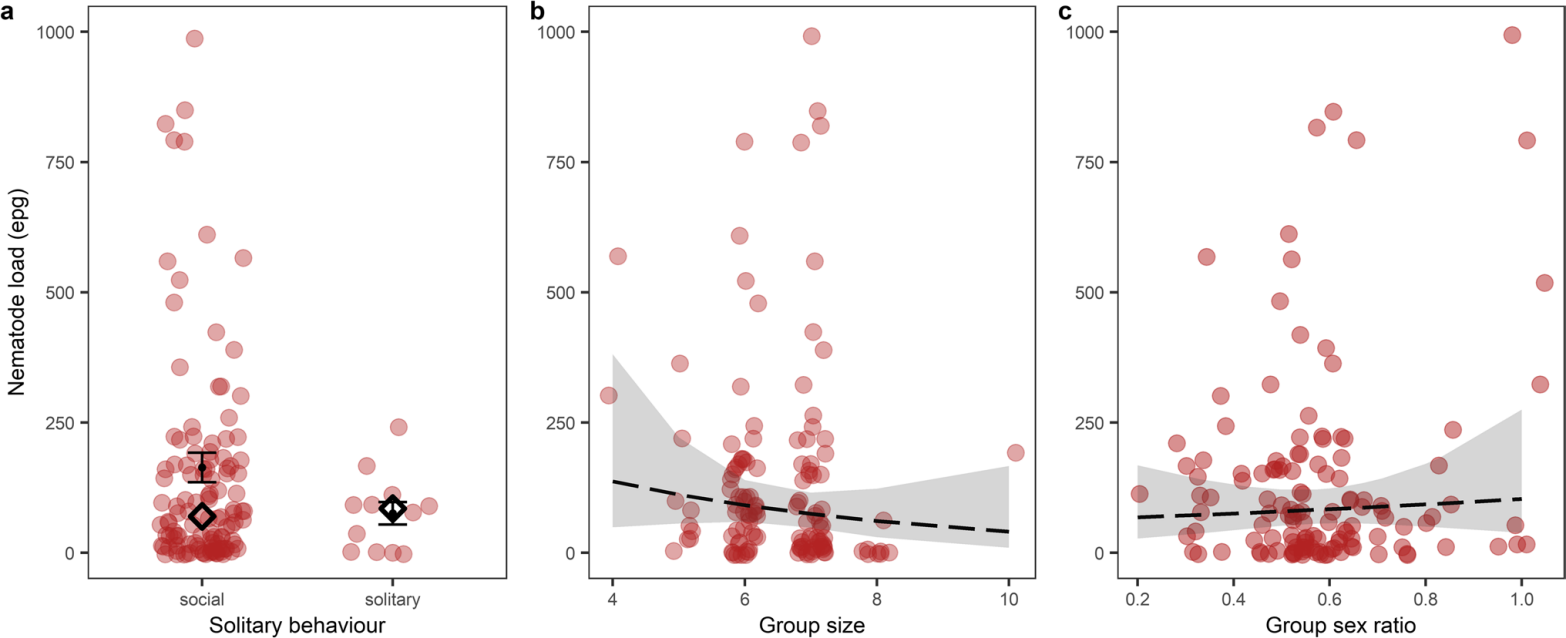
The social landscape of infection in Asian elephants highlighting no significant variation in infection, as estimated by faecal egg counts (FEC, in eggs per gram of faeces, epg) with differences in (**a**) solitary behaviour, (**b**) working group size, and (**c**) working group sex ratio. In total, 130 measures were collected from 71 individual elephants. Red points correspond to raw FECs, black points and error bars to mean and standard error FEC values, and black diamonds correspond to median FEC values. For (**c**), lines show predicted FECs, calculated in *R* using *ggpredict* (Lüdecke 2018), and shaded areas correspond to 95% confidence intervals. Plotted data is limited to FECs of 1000 epg, excluding one individual data point (2720epg)

We found strongyle (Nematoda; Strongylidae) and *Strongyloides* (Nematoda; Strongyloididae) type eggs within faecal samples, observed in different developmental stages. Nematode loads varied widely between individual hosts within our population; FECs were highly skewed (aggregation parameter *κ* = 0.272, variance:mean ratio ≥ 1), and ranged from 0 to 2720epg (mean ± SE = 156epg ± 26, median = 75epg). Additionally, seven elephants (~ 10% of hosts, 10 faecal samples corresponding to ~ 8% of FEC measures) had relatively high observable levels of egg shedding with FECs over 500epg (Nielsen et al. 2010), including only one elephant having a FEC much higher than 1000epg. We did not discard this value since this is a common pattern of nematode loads found in many species. Individual time since deworming ranged from 12 to 419 days of sampling (mean = 131 days), although ~ 80% of elephants had not been dewormed within 30 days prior to sampling (*n* = 105/130 individuals), and 65% of elephants had not received treatment for ~ 90 days before sampling (*n* = 84/130). We also found variation between individuals in their social measures; however, after accounting for variance from confounding factors, the differences in FECs were not associated with those in our tested social measures (Fig. 1, Table 1). We first tested for an influence of solitary behaviour on infection rates. Overall, elephants were mostly classed as social (118/130 answers relating to 62 different elephants—84 from females, 34 from males), and we recorded only 12/130 classifications of solitary for 9 different elephants (7 answers from females and 5 from males) from the social questionnaires. Overall, males were over twice as likely to be classified as solitary, which was recorded in 19% of all males studied (*n* = 5/26) in comparison to 9% of the total number of females (*n* = 4/45). However, while mean raw FECs were 46% higher for social elephants in comparison to solitary individuals (mean raw FEC ± SE = 164 ± 29 epg for social elephants vs. 76 ± 22 epg for solitary conspecifics, model estimate ± SE = 0.178 ± 0.456), these differences were not statistically significant (*χ*^2^ = 0.151, *p* = 0.658). Moreover, we found no evidence for any sex-specific differences in solitary behaviour influencing infection rates when including a sex*solitary behaviour interaction (Table 2, solitary behaviour: *χ*^2^ = 3.117, *p* = 0.077; 164 ± 37 mean raw epg for social females vs. 164 ± 38 epg for social males, 101 ± 30 epg for solitary females vs. 40 ± 25 epg for solitary males).

**Table 1:** Effect estimates from final models for predictors of faecal egg counts for each sociality measure, fitted with a negative binomial error structure and log link function. Working group ID number was included as a random effect. The intercept corresponds to FECs from elephants with 0 days since treatment, and that (1) displayed social rather than solitary behaviour, (2) lived in small working groups, and (3) lived in groups with a female:male sex ratio of 0. All models were fitted to 130 observations from 71 elephants. Significant effects (*p* < 0.05) are in bold

Sociality measure	Coefficient	Estimate	SE	*Z*	*Χ*^2^	*P*
1.Solitary behaviour	Intercept	1.255	0.283	4.440	-	-
	**Time since treatment (days)**	**0.007**	**0.001**	**4.528**	**20.427**	** < 0.001**
	Solitary behaviour (solitary)	− 0.178	0.456	− 0.390	0.151	0.698
		**Variance**	**SD**			
	Work group ID	0.800	0.894	-	-	-
2.Group size	Intercept	2.558	1.312	1.950	-	-
	**Time since treatment (days)**	**0.006**	**0.001**	**4.474**	**19.983**	** < 0.001**
	Group size	− 0.202	0.196	-1.030	1.044	0.307
		**Variance**	**SD**			
	Work group ID	0.762	0.873	-	-	-
3.Group sex ratio	Intercept	0.927	0.706	1.313	-	-
	**Time since treatment (days)**	**0.007**	**0.001**	**4.550**	**20.642**	** < 0.001**
	Group sex ratio	0.519	1.904	0.474	0.221	0.639
		**Variance**	**SD**			
	Work group ID	0.764	0.874	-	-	-

**Table 2:** LRT and *P* values for comparisons of models (as described in Table 1) but including a fixed term for sex and a social measure*sex interaction term, and replicate models consisting only of main effect terms. All models were fitted to 130 observations from 71 elephants

Sociality measure interaction	*Χ*^2^	*P*
Solitary behaviour*sex	3.117	0.077
Group size*sex	2.475	0.116
Group sex ratio*sex	0.078	0.780

We next investigated associations between FECs and the size of working groups. In our population, elephants lived in groups of varying size (range = 4–10, mean = 6.5, median = 7). After accounting for variance from treatment and from repeated measures, our results indicate that infection is lower for elephants in larger groups (model estimate ± SE − 0.202 ± 0.196). As with solitary behaviour, this difference was not significant (*χ*^2^ = 1.044, *p* = 0.307) when controlling for other contributing factors. Furthermore, we found no evidence that egg shedding in males and females differs respectively in response to more or fewer group members when testing for an interaction between FEC and group size (Table 2, *χ*^2^ = 2.475, *p* = 0.116).

Finally, we determined the effect of group female:male sex ratio on infection dynamics. Sex ratios of the different working groups ranged from 0.2 to 1 (mean = 0.58, median = 0.57), where an increasing ratio equals an increasing proportion of females within a group (1 = all-female group). FECs increased with increasing sex ratio, i.e. in groups with proportionally more females (model estimate ± SE 0.519 ± 1.904). However, as with our other sociality measures, we found no significant association overall between working group sex ratio and later infection status in our host population (χ^2^ = 0.221, *p* = 0.639). Again, we found no evidence of sex-specific differences of group sex ratio on later infection rates when including an interaction term with sex (Table 2, *χ*^2^ = 0.078, *p* = 0.780). All these results are robust when data are limited to FECs and social data collected within 1 day.

## Discussion

Here, we investigated associations between three specific aspects of host sociality—individual solitary behaviour, group size, and group sex ratio—and infection by gastro-intestinal nematodes (strongyles and *Strongyloides* spp*.*), in a long-lived mammal with a complex social structure, the Asian elephant. Our study population consisted of 71 semi-captive Asian elephants of mixed sexes and ages, grouped into working units of various sizes (4–10 individuals) which spent diurnal hours together, but were able to display natural nocturnal roaming, socialising, and mating behaviours with other semi-captive and local wild conspecifics. We argue that this difference in social structure to their natural matriarchal herds allows for a ‘natural experiment’ to observe how infection is linked to the studied measures of sociality across different social frameworks. Controlling for other known confounding factors, we show that infection was not associated with any investigated measure of sociality, and that this finding was conserved across both males and females. Generally, while our results contradict our expectations, they support the argument that the parasite-related costs of sociality may vary in magnitude, are not linear, and do not operate solely in one direction. Recent studies highlight a more complex picture—that the extent of parasite-related costs, or the severity at which they are felt, may hinge on other aspects of host ecology, for example individual life history (Ezenwa et al. 2016), differences in dominance hierarchies within a social unit (Smyth and Drea 2016), or the degree of modularity or subgrouping within a population (Nunn et al. 2015). As such, there is increasing support for an ‘expanded view’ that infection, or the fitness costs thereof, may in fact be minimised through socially promoted resistance and/or tolerance pathways (Ezenwa et al. 2016).

While we found that males were nearly twice as likely to display solitary behaviour, neither individual solitary nor social behaviour influenced infection by strongyle or *Strongyloides* nematodes for any individual in our sample. When comparing across systems, infection measures are higher for social animals than those for solitary ones (Ezenwa et al. 2016). However, crucially, studies often compare the effects of infection, and selection for solitary versus gregarious behaviour, across species, rather than observing intraspecific variation within groups. Consequently, much less is known of how infection costs relate to variation in individual solitary behaviour within populations, which is an oversight considering that sociality is not homogenous within species. For example, in a number of ‘social’ species, individuals may realistically exhibit behaviours over a spectrum from more solitary to more social, with behavioural tendencies varying with other traits such as age and sex, as found in e.g. elephants, hamadryas baboons (*Papio hamadryas hamadryas*: Schreier and Swedell 2012), and Western lowland gorillas (*Gorilla gorilla gorilla*: Racevska and Hill 2017). Finer-scale analysis has found elephant societies show multilevel organisation and fission–fusion dynamics, with populations varying in their degree of modularity, hierarchical levels, and the extent to which these are nested (de Silva and Wittemyer 2012; Nandini et al. 2018). There is also evidence that individuals maintain long-term affiliate relationships alongside ephemeral associations with conspecifics (de Silva and Wittemyer 2012), suggesting that interactiveness of both males and females to conspecifics both within and outside social units may not be temporally stable, and that tendencies of social versus solitary behaviour may change over time. Therefore, while broader classifications of sociality (i.e. as with our binary measure of solitary behaviour) are still highly valuable, especially from lesser-studied taxa, such methods may not capture the sufficient detail needed to elucidate how potential parasitism constrains finer structural contexts, for example, establishing how infection changes with increasing social contacts, or frequency or quality of interactions with other group members. While recent studies offer initial insights as to how infection operates with varying modularity in animal populations (Sah et al. 2017), there is still great scope for future studies to investigate how infection costs are incurred, and alleviated, over multi-level societies with complex coalitions, such as those seen in elephants and primates.

Group size remains one of the most widely studied predictors of parasite risk as a disease cost of sociality. Increases in group size are coupled with higher spatial–temporal concentration of potential hosts and more frequent conspecific interactions, which facilitates increased transmission risk and exposure to infective agents (Altizer et al. 2003; Rifkin et al. 2012). However, we found no evidence to support an association between nematode infection and group size. In fact, after accounting for confounding covariates, infection was overall lower for hosts in larger groups. Our results are surprising considering other studies on contagious and environmentally transmitting parasites, like gastro-intestinal nematodes, mostly show positive associations, although the size of this effect is smaller in mammalian hosts than that in birds (Rifkin et al. 2012). Instead, our results were more comparable to those on searching parasites, which are mobile enough to move between host aggregations, e.g. ectoparasites such as lice, ticks, and fleas (Rifkin et al. 2012). However, nematode motility is unlikely to substantially influence sociality–infection dynamics in our system. While elephant nematodes exhibit host-seeking behaviour in their infective stage after their third larval moult (Fowler and Mikota 2006), the distances travelled are miniscule in comparison to the roaming distances and range sizes of their elephant hosts (see Gang and Hallem 2016). While previous studies have noted a lack of association between infection and group size (Côté and Poulin 1995), it remains a relatively rare observation, possibly explained by publication bias (Rifkin et al. 2012), with group size weakly predicting parasite risk across most taxa (Côté and Poulin 1995; Rifkin et al. 2012). Interestingly, group size does not predict parasite intensity in a range of other mobile hosts (Patterson and Ruckstuhl 2013), including for other herbivore–strongyle systems, such as Grant’s gazelle (*Gazella granti*), buffalo (*Syncerus caffer*), impala (*Aepyceros melampus*), and eland (*Taurotragus oryx*) (Ezenwa 2004). One possible explanation is that mobile hosts gain resistance benefits from living in larger groups, as individuals that travel over larger ranges spend less time overall within any given area and thus reduce exposure in parasite-contaminated areas (Côté and Poulin 1995). While our study elephants work within designated forest areas during the day, they are able to roam, unsupervised, over larger distances at night—a behaviour that may mitigate the parasite infection risk incurred through their diurnal work grouping.

The focus on group size as a primary predictor of infection costs is a relatively simple view of the linkage between sociality and disease, as group-living species display huge variation in both the size and structure of social landscapes. Group sex ratio, and how this factors into disease costs, is a relatively overlooked aspect of group living, which is an oversight considering how widely observed sex biases are in infection in wild systems (Wilson et al. 2002). In mammals, parasite infection intensity is often higher for adult males than females (Giery and Layman 2019)—a consequence of hormone-mediated differences in resource allocation trade-offs between immunity and reproduction (Hamilton and Zuk 1982; Folstad and Karter 1992; Stearns 1992). Curiously, a previous work has highlighted a lack of sex-biased parasitism within the Myanmar timber elephant population, with males and females harbouring similar nematode loads across a longitudinal study period (Lynsdale et al. 2020), despite males incurring higher mortality cost of parasitism. The close proximity between individuals in mixed-sex working groups may increase transmission between males and females, possibly concealing inherent sex-specific differences in susceptibility. Yet, as with group size, infection was not associated with variation in sex ratio of working groups, suggesting that social framework does not mask differences in nematode loads between males and females in this system. Despite this, group sex ratio should be regarded as an important potential driver of associations between sociality and infection in other systems, particularly where sex biases in infection rates are observed.

The reasons underlying the absence of associations between infection and solitary behaviour therefore remain largely obscure. A possible explanation is that for reproductive-age adult elephants, nematodes are less pathogenic in comparison to e.g. bacterial and viral infections that severely impacting host survival (Fowler and Mikota 2006), or that loads do no reach critical thresholds, exacting low costs to individual morbidity and relatively weak selection pressures on sociality. However, this seems unlikely. Preliminary work has shown that observed nematode infection significantly reduces individual health and condition, as measured by white blood cell counts and liver function (Franco dos Santos D., unpublished). Moreover, our host population displays high heterogeneity in infection; nematode loads can reach exceedingly high burdens (> 4000epg), beyond ‘high’ shedding veterinary thresholds for other non-ruminant hosts (Nielsen et al. 2013), but only for specific demographic groups (Lynsdale et al. 2020). In particular, juveniles show both elevated loads estimated via FECs (Lynsdale et al. 2020), and historically, along with adult males and non-reproductive females, suffer from increased mortality as a result of parasitism (Lynsdale et al. 2017). This, coupled with the fact that timber elephants live in mobile working units without strong competition or dominance hierarchies, may instead mean that the strongyle and *Strongyloides* nematodes either do not present large sociality costs in this system or that these are mitigated by the social health benefits of group living more than in other host taxa. The known variation in FECs observed across different elephant ages could provide an explanation for the lack of a link between our social measures and FECs; as acquired immune function varies across vertebrate lifespans, strong age-specific susceptibility effects may override effects of sociality in hosts, which have been previously exposed to nematodes. However, in this study, we account for age-specific variation in FECs by including host age in our analysis, allowing us to reliably detect any strong associations with sociality measures. However, it should still be noted that other individual differences in infection and exposure profiles may potentially mask weaker associations between the sociality measures investigated and FECs. Another unexplored avenue of interest is self-medicating behaviour, as observed in numerous primates (Neco et al. 2019), and in Asian elephants (Greene et al. 2020). For example, red colobus monkeys (*Procolobus rufomitratus tephrosceles*) increase their consumption of fodder with known anthelminthic properties, such as certain barks and *Albizia* spp*.* plants, during periods of increased shedding of whipworm (*Trichuris* spp.) eggs (Ghai et al. 2015). In elephants, specific plant consumption is thought to relate to self-medication behaviour for certain medical ailments, including parasitism, according to local human mahouts and knowledge holders (Greene et al. 2020). Behavioural switches to sole consumption of clay rather than vegetative matter are also noted by Asian elephants’ mahouts during monsoon months, which is thought to aid in expelling established gastro-intestinal parasite infections (Greene et al. 2020). As foraging decisions can be transferred through cultural transmission in primates (Horner et al. 2006), Ezenwa et al. (2016) propose that social living and large group sizes may promote self-medicating selective foraging as a behavioural mechanism for parasite resistance. Studies have also suggested that this strategy may particularly benefit larger, longer-lived species (Neco et al. 2019), such as Asian elephants, which are also generalist browsers and graze feeders and have been known to vary their diet in response to environmental change (Sukumar 2003).

Our results provide a reliable insight into whether strong social–infection associations exist by utilizing a centralized keeping system in a rarely studied host system—semi-captive timber elephants in Myanmar. The elephant mahouts have a detailed knowledge of their elephant’s behaviours and collect the elephants in the morning from the forest meaning that they are aware of whether elephants are exhibiting solitary behaviour, and whether they are grouped with the same group members or other working individuals, during part of the unsupervised period. However, it should be stressed that we cannot account for variance from any nocturnal social interactions and individual differences in foraging activity (e.g. Parker et al. 2020). While data on nocturnal activity of elephants is limited, and largely focused on fully captive systems, there is some evidence that elephants may be stationary for large periods of the night (Wilson et al. 2006; Lukacs et al. 2016), and that activity depends on age and access to outside areas (Evison et al. 2020) suggesting that most social activity takes place during diurnal hours. The measures of elephant sociality used in our study might have been too broad to capture any potential weak infection–sociality associations present in our study population or actually not capture specific social–infection mechanisms. Therefore, we cannot exclude the possibility that finer-scale measures than those investigated here might show a different picture. Data on social network dynamics and characteristics might provide the needed fine-scale measures. Specific network components, such as connectivity or centrality within the social network, can relate to transmission dynamics (Rimbach et al. 2015). Unfortunately, the qualitative nature of our questionnaire data does not allow assessing those network characteristics in detail. Other confounding factors, such as the distribution of high-shedding individuals sharing the work areas with focal individuals and the effects of season on infection dynamics, should be noted. While faecal egg counts are moderately repeatable within hosts of our study population (Lynsdale et al. 2020), we do not know how genetic components contributed to high-shedding behaviour and hence we cannot directly control for this factor. However, including individual identity as a random factor in our analyses should help mitigate this bias to some extent. Regarding seasonal effects, this study used data collected only during one season (dry season) and hence we can exclude seasonal biases on our results, but to complete our understanding of infection–sociality associations they should also be investigated in other seasons (monsoon and cold season). Finally, although our study population shares more characteristics with wild elephant populations than fully captive populations, we suggest that our results should be treated with care when comparing to truly wild populations. The potential impact of human handling on social behaviours and group composition of our study elephants and the strong effect of regular anthelmintic treatments should be kept in mind when interpreting our results. However, some of these confounding factors constitute general challenges to studies investigating infection–sociality associations in the wild, and we were able to control for several other confounding factors of susceptibility such as age. Thus, we suggest that our findings are still a valuable addition to the literature, with very few other studies using adequate sample sizes and providing insights into the social infection dynamics of extremely long-lived terrestrial mammals.

In conclusion, our results further highlight the need for a general push towards placing social infection dynamics clearly in specific contexts, and the necessity for more studies investigating different facets of sociality from a diverse range of host-parasite systems, to inform broader meta-analyses. It is becoming increasingly clear that the relative costs of disease are determined by a number of social traits, and their organisation across different social landscapes, acting in synergy; in essence it is ‘more than just a numbers game’ (Nunn et al. 2015). Consequently, there is a growing emphasis on establishing how the sociality–disease nexus varies across and within a range of taxa, with elephants presenting a much-needed comparison to other long-lived, complex mammal societies. Here, we highlight the need for finer-scale studies, establishing how sociality is limited by, mitigates, or protects against infection in different ecological contexts, to fully understand the mechanisms underlying these pathways.

## Supplementary Information

Below is the link to the electronic supplementary material.

Supplementary file1 (R 7 KB)

Supplementary file2 (CSV 7 KB)

## Data Availability

Data and code are available as electronic supplementary material.
